# Expression of Chemoresistance-Related Genes and Heat Shock Protein 72 in Hyperthermic Isolated Limb Perfusion of Malignant Melanoma: An Experimental Study

**DOI:** 10.1155/2010/138758

**Published:** 2010-06-20

**Authors:** C. Knorr, J. O. Pelz, J. Göhl, W. Hohenberger, T. Meyer

**Affiliations:** ^1^Department of Surgery, University of Erlangen-Nürnberg, 91054 Erlangen, Germany; ^2^Department of Surgery, Klinikum Ansbach, Germany

## Abstract

Hyperthermic isolated limb perfusion (HILP) is considered an established treatment for multiple locoregional intransit metastases in malignant melanoma of the extremities. Various mechanisms such as the expression of chemoresistance genes and heat shock proteins by the tumor may be responsible for varying response rates and locoregional recurrences of the treatment. The aim of the experimental animal study was to investigate the direct impact of HILP on such mechanisms of resistance. Tissue temperature, administration of the cytostatic drug, and duration of perfusion were varied. Expression of the chemoresistance genes mdr1, mrp1, mrp2, and lrp and of heat shock protein 72 (HSP72) in the tumor tissue was analysed using RT-PCR and western blot analysis. The untreated SK-MEL-3 tumor expressed mdr1, mrp1, and lrp, but not mrp2. Neither variation of temperature, administration of the cytostatic drug, nor duration of perfusion changed the expression of this “resistance pattern”. In contrast to the cytostatic drug, hyperthermia causes a persistent induction of HSP72. Both observations could offer a potential explanation for failure of HILP in malignant melanoma.

## 1. Introduction

Hyperthermic isolated limb perfusion (HILP) is regarded as an established therapy for locoregional intransit metastases of malignant melanoma of the extremities. Isolation of the limb by extracorporeal circulation, that is, by using a heart-lung-machine, enables administration of high doses of cytostatic drugs without relevant systemic side effects. Simultaneous heating of the limb up to temperatures of 40.0°C to 41.0°C intensifies the tumortoxic action of the drug [1]. Although HILP represents a form of regional high-dose thermo-chemotherapy, partial response or treatment failure is observed in 25–50% of patients. Furthermore, up to 50% of patients with previous complete remission following HILP will undergo relapse on the treated limb, usually after a median of 9–12 months [2–4].

Among the main causes for failure of treatment is the resistance of tumor cells against the action of cytostatic drugs, which is based on the expression of certain genes such as the multidrug resistance gene (*mdr1*), coding for the p-glycoprotein (*p-gp*) [5]. The gene product functions as an energy-dependent membrane-bound transport protein for hydrophobic, cytotoxic agents such as anthracyclines, epipodophyllotoxins, vinca alcaloids and taxol, thus lowering their intracellular concentration by drug efflux [6]. Meanwhile other chemoresistance-related proteins like the multidrug resistance protein MRP1 and its isoform MRP2 (canalicular MRP, cMRP; canalicular multispecific organic anion transporter, cMOAT) and the lung resistance-related protein (LRP) have been identified [7, 8].

Furthermore, the expression of various heat shock proteins (HSP) has been associated with resistance of tumor cells to thermal and chemical damage [9]. The function of the different families of heat shock proteins is complex and has not yet been understood in full detail. One of the proteins highly inducible under stress conditions is the 72 kD heat shock protein (HSP72), a member of the HSP70 family. According to their function as molecular chaperones [10], a cell-protective effect is ascribed to these proteins after impairment of normal cell physiology by toxic stimuli such as heat, ischemia, and inflammation, although meanwhile proapoptotic cascades have also been linked with the induction of HSP [11]. Tumor cells respond to HSP-mediated stress in the same way and can escape the action of tumortoxic therapies.

This has prompted us to investigate the direct impact of HILP on molecular mechanisms of resistance of malignant melanoma in an experimental study. A set of well-characterized chemoresistance-related genes (i.e., *mdr1*, mrp1, mrp2, lrp) and HSP72 were selected for targets of the effects of HILP for purposes of the study.

## 2. Materials and Methods

### 2.1. Cell Lines and Animals

KB-3-1 cell-line (DSZM, Braunschweig, FRG): as a derivative of the human cervix carcinoma cell line HELA, it is used in multiple drug resistance studies giving rise to drug-resistance mutants (e.g., KB-V1, resistant to vinblastine). KB-3-1 is negative for *mdr1* mRNA expression and served as a negative control in the *mdr1* detection experiments.

SK-MEL-3 cell-line (American Type Culture Collection, Rockeville/Md., US): cell line established from a lymph node metastasis of malignant melanoma in a 42-year-old Caucasian woman. A cell suspension of SK-MEL-3 was injected subcutaneously on athymic nude mice (NMRI nu/nu) to create a solid tumor. Small particles (approx. 5 mm^3^) of the tumor were implanted subcutaneously on the right hind limbs of the study animals by means of small skin incision.

Animals: for experimental limb perfusions eight-week-old athymic nude rats (Rowett rnu/rnu) weighing 200–250 g and bearing the SK-MEL-3 xenograft on their right hind limbs were used. Perfusions were performed when the tumor reached a size of approximately 25 mm².

### 2.2. Drug

10 mg of dry substance vinblastine sulfate (Velbe, Lilly, Bad Homburg, FRG) were dissolved in 10 ml 0.9% NaCl. For experimental perfusions, 25 *μ*g vinblastine/15 ml priming volume were injected as a fractionated bolus over 2 minutes into the arterial line. Previous dose-finding studies had shown that this concentration was well tolerated by the animals and led to complete tumor regression in single animals. Vinblastine was chosen because of its cytotoxic effect on melanoma cell lines, which has been proven in former experimental perfusion studies [12]. Furthermore, vinblastine is one of the substrates that is eliminated by the p-glycoprotein pathway.

### 2.3. HILP Treatment and Surgery

A modified miniature equipment, first described by Nagel et al. 1987 [13], for rat limb perfusion was used. Our technique of experimental perfusion has been described in detail earlier [1].

In brief, after induction of anesthesia, the animal was fixed in a supine position on a cork-coated operation table. A vertical skin incision was performed in the right groin and the inguinal ligament was divided. The external iliac and femoral vessels were exposed by retracting the peritoneal sac in craniomedial direction. After heparinization with 1000 IE/kg body weight, all collateral vessels were temporarily clipped to reduce systemic leakage. After central clamping of the external iliac artery and vein, both vessels were cannulated under microscopic control (G 24 and G 20 catheters, resp.). The catheters were connected with the perfusion system and the extracorporeal circulation was started with a flow rate of 0.4 ml/kg body weight. A subcutaneous temperature probe (Yellow Springs Instrument Co., US) was placed near the tumor for continuous registration of tissue temperature (Stöckert, FRG).

The extracorporeal circulation was achieved using a Masterflex roller pump (Reichelt, FRG) with two pump-heads running synchronously on a single axis for the arterial and venous line. Venous blood from the tumor bearing limb was directed towards a specially constructed gas dispersion oxygenator (Lettenbauer, FRG) made of high-quality acryl with foam material taken from commercially available oxygenators. The oxygenated blood was then redirected into the limb. Tygon tubing (Reichelt, FRG) with an internal diameter of 0.8 mm was used for circulation of the perfusate. 3-way taps in the arterial and venous line allowed injection of the drug, measurement of blood gas analysis, and registration of perfusion pressure (if necessary). Hyperthermia was induced by an externally adjustable infrared light source centered onto the tumor area.

### 2.4. Treatment Schedule

Apart from the analysis of the untreated tumor (*n* = 3), four groups with 12 animals each were constituted. Group I comprised normothermic perfusions (tissue temperature, 37–37.5°C) without vinblastine, group II normothermic perfusions with vinblastine (25 *μ*g/15 mL perfusate), group III hyperthermic perfusions (tissue temperature 40.0-41.0°C) without vinblastine, and group IV hyperthermic perfusions with vinblastine (25 *μ*g/15 mL perfusate). Each group (*n* = 12) was divided into four subgroups (*n* = 3) with variation of perfusion time. The duration of perfusion was 30, 60, and 90 minutes. In the fourth subgroup, animals survived 5 hours after a 60-minute perfusion to evaluate early posttreatment effects. After the scheduled perfusion time, the tumor was excised and instantly frozen in liquid nitrogen.

### 2.5. cDNA-Synthesis and Reverse Transcriptase-Polymerase Chain Reaction (RT-PCR)

Gene expression was monitored using RT-PCR method. Total cellular RNA was prepared from 30 mg tissue samples using the QIAGEN RNeasy-kit. cDNA was synthesized with 1 *μ*g of total cellular RNA and 50 pmol of random hexanucleotide primer (Perkin Elmer) in 20 *μ*l of a solution containing 5 mM MgCl2, 1xPCR-buffer II (Perkin Elmer), 500 *μ*M each dNTP, 20 U Rnase Inhibitor (Perkin Elmer), and 50 U MuLV Reverse Transcriptase (Perkin Elmer). The reaction was performed using a Trio-Thermoblock (Biometra) with the following program: 21°C for 10 minutes, 42°C for 60 minutes, and denaturation at 95°C for 5 minutes. Water was added to a final volume of 50 *μ*l and cDNA was stored at −20°C before it was used. RT-PCR was performed with 5 *μ*l of cDNA template in a 50 *μ*l reaction containing 0.15 mM MgCl_2_, 10 mM Tris/HCl pH 8, 3; 50 mM KCl, 50 pmol 5′-primer, 50 pmol 3′-primer, 2.5 U Ampli-Taq Gold (Perkin Elmer). After denaturation at 95°C for 5 minutes, PCR was performed as follows: 45 seconds 94°C, 45 seconds 60°C, 90 seconds 72°C for 34 cycles, with an additional elongation step at 72°C for 3 minutes, PCR products were analyzed on a 2.5% Agarose gel. PCR-primer sequences were *mdr1*-51 5′-GTT.CAA.ACT.TCT.GCT.CCT.GAG-3′; *mdr1*-31 5′-ACC.CAT.CAT.TGC.AAT.AGC.AGG-3′; mrp1-51 5′-ACC.GGA.GGA. TGT.TGA. ACA.AG-3′; mrp1-31 5′-AAT.GCG.CCA.AGA.CTA.GGA.AG-3′; mrp2-51 5′-CTG.CCA.TAA.TGT. CCA.GGT.TC-3′; mrp2-31 5′-CTG.GTT.GAT.GAA.GGC.TCT.GT-3′; lrp1-51 5′ TGG.AGC.CAT.CGG.TGA.TGA.GG-3′; lrp1-31 5′-TCT.GAG.CAT.GGC.CGT.GGA.GA-3′.

The expression of GAPDH was used as a positive control in each RT-PCR analysis (primer sequence: GAPDH-31 5′-CCA.TCA.CCA.TCT.TCC.AGG.AG-3′, GAPDH-51 5′-CCT.GCT. TCA.CCA.CCT.TCT.TG-3′).

### 2.6. Western Blot Analysis

HSP72 was monitored by western blot analysis. In addition, the gene product of *mdr1*, the p-glycoprotein, was measured representatively for all chemoresistance genes by the western blot method to detect the transcriptional activity of the gene. Total protein samples were prepared from 50 mg tissue samples, adding 100 *μ*l of Tris/HCl pH 7.5, 0.1% SDS, using a Micro-Dismembranator for 60 seconds, 2000 rpm. 500 *μ*l of 10 mM Tris/HCl pH 7.5; 1.0% SDS was added to the cell suspension and boiled for 10 minutes. After centrifugation for 10 minutes at 13.000 rpm, supernatant was collected and the protein content was measured by BioRad-Protein-Assay. 25 *μ*g of protein was applied per lane to a 7.5% SDS-PAGE-gel for *MDR1 *protein analysis and 12% SDS-PAGE gel for HSP72 analysis. The gel was blotted on a nitrocellulose membrane with 0.8 mA/cm² gel for 1.5 hours using a semidry gel-blotting apparatus. The membrane was blocked using a 5% solution of blocking-reagent (Amersham) in TBS-T, for one hour at room temperature. Protein detection was performed with “Monoclonal Anti-72 kDa heat shock protein” (Amersham Life Science) and “*mdr* (P-glycoprotein) (Ab-1)” (Calbiochem). Incubation was performed at room temperature for one hour. After three washing steps, secondary antibodies, antimouse-Antibody (Amersham), and antirabbit-antibody (Amersham), respectively, were applied. Antibody detection was done with ECL-System (Amersham Life Science).

### 2.7. Evaluation of HSP72 Expression

HSP72 expression was evaluated semiquantitatively by densitometric measurement and band analysis with the imaging software TINA (Raytest). Bands were compared to a dilution series of protein samples to calculate fold expression. Pixel areas of the bands in each group (*n* = 3) were averaged and related to the mean pixel area of the untreated tumor which represented the value 1.

## 3. Results

All experimental perfusions could be performed as planned except the 90-minute hyperthermic perfusions (with and without vinblastine) which were not tolerated by the study animals (intraoperative exitus). Thus, the number of experimental perfusions actually performed totalled 42.

### 3.1. Expression of Chemoresistance Genes

*Mdr1*, mrp1, and lrp were constantly expressed in the untreated SK-MEL-3 tumor, whereas mrp2-mRNA could not be detected in any of the untreated tumor samples. This pattern of expression was neither remarkably influenced by the application of hyperthermic temperatures nor by the addition of the cytostatic drug vinblastine, nor did variation of the length of perfusion appreciably change expression of the studied chemoresistance genes. In single-tumor samples, a temporary suppression of mrp1 in groups I, II, and III was observed as well as a passing induction of mrp2 in groups II, III, and IV. No later than 5 hours after the 60-minute perfusion time, the original pattern of expression was restituted as detected in the untreated tumor. Detection of the p-glycoprotein by western blot analysis corresponded to the results of *mdr1* expression by RT-PCR (data not shown). Variability in expression of single genes within the subgroups at different times was low: there was a congruence of expression in 51 of 60 subgroups (85%). Deviations within a group at a defined time could correspond to a true therapeutic effect, but could also be caused by the immanent variability of measurements in biological systems. A fundamental change of the intrinsic “resistance pattern” following HILP could not be observed in the SK-MEL-3 tumor.

### 3.2. Expression of Heat Shock Protein 72

The SK-MEL-3 tumor constitutively expressed HSP72. At normothermic tissue temperatures (group I), only a temporary 1.5-fold induction in comparison with the base line of the untreated tumor was observed after 30 minutes. The administration of vinblastine under normothermic conditions (group II) was associated with an approximately 2.5-fold increase of HSP72 production, which dropped to a 1.5-fold rate of expression after 90 minutes. 5 hours postoperatively (60-minute perfusion time), the level of the untreated tumor was reached again. Hyperthermia alone caused a delayed increase of HSP72 expression by factor 1.5, which was kept even for 5 hours after the perfusion had been terminated (group III). Against the expectations, only a 1.5-fold induction was measured in combined application of hyperthermia and vinblastine (group IV), though it was observed after only 30 minutes of perfusion and persisted as long as 5 hours after the perfusion had been terminated.

## 4. Discussion

Chemoresistance of malignant melanoma is a well-known phenomenon. Even with the most effective cytostatic drug, that is, dacarbazine, remissions are reported to be only as high as 14–33% in the metastasized stage of disease [14]. One of the best-characterized mechanisms of chemoresistance is the *mdr1* gene, but some studies have shown that it is rarely overexpressed in malignant melanoma [15–18]. On the other hand, increased expression of the chemoresistance-associated transport proteins MRP and LRP was found in melanoma cell lines as well as in primary and metastatic melanoma that could explain resistance towards lipophilic, natural compounds, but not towards alkylating agents [16].

The SK-MEL-3 tumor constitutively expressed *mdr1*, mrp1, and lrp. Because coexpression of several mechanisms of resistance is related to prognosis [18–20], the SK-MEL-3 tumor can be considered as a relatively resistant variant with an unfavourable susceptibility. This is supported by the fact that the tumor was isolated from a lymph node metastasis from a patient that had been pretreated with Methyl-CCNU. Cytostatic pretreatment can also be correlated with the presence of *mdr1*-mRNA and p-gp in the SK-MEL-3 tumor because the incidence of mdr1 expression has been observed to increase following previous chemotherapy, for example, in acute myeloic leucemia, plasmocytoma, or osteosarcoma, and adversely influences adversely remission rate and prognosis of relapsed tumors [21]. In contrast, Schadendorf et al. [17] did not find an enhanced induction of p-gp in vindesine- and cisplatin-pretreated metastasic melanoma.

The pattern of expression of chemoresistance genes was not changed by hyperthermic isolated limb perfusion and seems irreversibly determined. Despite high concentrations of the cytostatic drug and the exposition towards hyperthermic temperatures, only temporary effects on gene expression, that is, mrp1 (suppression) and mrp2 (induction) could be observed. These were limited to the action of treatment. Apparently, even high-dose thermochemotherapy is not able to alter transcriptional activity of chemoresistance genes. Although the results may only apply to the SK-MEL-3 tumor and the number of study animals in each subgroup was rather small to draw a final conclusion, preliminary results in melanoma patients treated by HILP underline this observation [22].

Should this be confirmed in further experimental and clinical studies, it would be desirable to define the resistance pattern preoperatively for better evaluation of the response to HILP or adjusting the drug regimen more individually to each patient, that is, a calculated choice of agents according to the individual resistance pattern. Furthermore, an additional administration of blocking agents for inhibition of resistance genes, that is, inhibition of *mdr1* by verapamil- or ciclosporine-derivatives can be imagined. There are first promising clinical experiences with such modulators of chemoresistance in the systemic treatment of refractory non-Hodgkin lymphoma and plasmocytoma [23, 24], but side effects of the therapy were limiting. The regional action of HILP should offer an ideal indication for these substances because systemic side effects of the therapy are negligible.

In the literature, increased expression of HSP (HSP27, HSP70) plays an important role in reduced sensitivity towards cytostatic drugs [25–27]. A direct correlation of chemoresistance and heat shock has been postulated by Chin et al. [28, 29] and Kioka et al. [30] who observed an amplification of *mdr1*-mRNA together with an enhanced resistance of tumor cells in vitro towards vinblastine after heat exposure. The explanation that was given for this result was the presence of heat shock-responsible consensus elements in the *mdr1*-promotor region. In melanoma cells not only a chemically and heat-induced expression of HSP (HSP72) has been demonstrated [31–34], but also a constitutive overexpression that was independent of stress (HSP27, HSP72, HSP90), as was the case in the SK-MEL-3 tumor. Again, constitutive expression of HSP was associated with an advanced tumor stage and amplified resistance towards thermal and cytostatic drug damage [35–37].

The cellular response to stress, that is, the increase in transscriptional activity of HSP, takes place within minutes [38]. In vitro studies in melanoma cells showed the level of HSP expression to be dependent on time and temperature, with the maximal rate of synthesis at 42°C and 9 hours exposure reaching a steady state with levels not much higher than constitutive expression following continued exposure [31].

These in vitro results were also obvious in the in vivo tumor model which was presented. The sensitive stress reaction is recognized by the fact that even operative manipulation alone (normothermic perfusion without vinblastine) was associated with a raised HSP synthesis, probably due to limb ischemia during the clamping of the vessels for cannulation. The administration of vinblastine in normothermic perfusions resulted in peak values of HSP72 synthesis after 30 minutes (factor 2.5), which dropped to the base line of the untreated tumor not later than 5 hours after 60-minute perfusion, maybe as a consequence of *mdr1*-mediated drug efflux of vinblastine. The increase of HSP72 expression in the tumor tissue during hyperthermic temperatures was clearly evident, but with a delayed maximum for hyperthermia alone (60 minutes) in comparison with the combination of hyperthermia and vinblastine (30 minutes). Hyperthermic temperatures led to a persistently enhanced synthesis of HSP72 in comparison with normothermia. This continued for hours after the operation. The reason why the combination of both stress factors (cytostatic drug and hyperthermia) had a smaller effect on the level of HSP72 expression remains unclear, but a temporary paralysis of cell function including protein synthesis with gradual recovery could be discussed. The changes in the rate of synthesis by a factor of 1.5 to 2.5 may be less impressive than in an in vitro setting, but the vascularized structure of a solid tumor undoubtedly represents a more complex system so that despite standardized experimental conditions, biologic effects are more difficult to assess. The constitutive expression of HSP72 in SK-Mel-3 tumor may add to this observation.

## 5. Conclusion

As has been reported earlier [1, 39, 40], the proposed experimental model, that is, performance of miniaturised limb perfusion in nude rats with implanted melanoma xenografts simulating the clinical setting proved to be suitable for the investigation of the question of how well-characterized mechanisms of resistance are directly influenced by HILP. On the one hand, the inherent, irreversible pattern of chemoresistance genes and the induction of HSP in the tumor itself during treatment offer a possible explanation for a primary (no or partial response) or secondary failure (early local recurrence) of HILP. On the other hand, there is clinical evidence that apart from a resistant cell phenotype there are additional factors such as tumor burden, which are important for response or resistance of the tumor towards therapy [22].

## Figures and Tables

**Figure 1:**
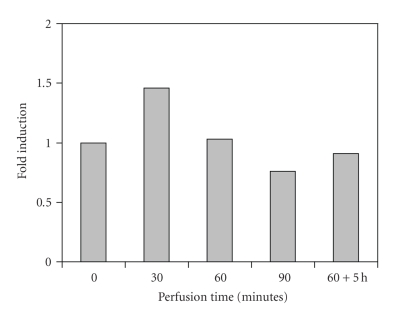
Group I (*n* = 12). HSP72 expression in normothermic limb perfusion without cytostatic drug.

**Figure 2:**
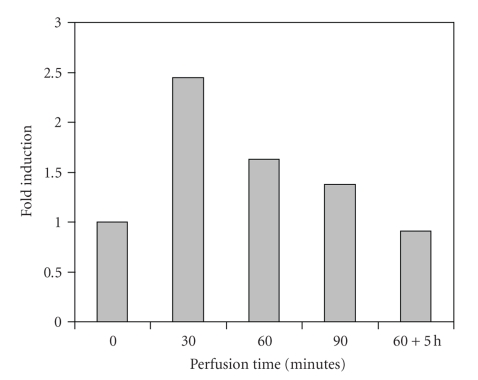
Group II (*n* = 12). HSP72 expression in normothermic limb perfusion with vinblastine.

**Figure 3:**
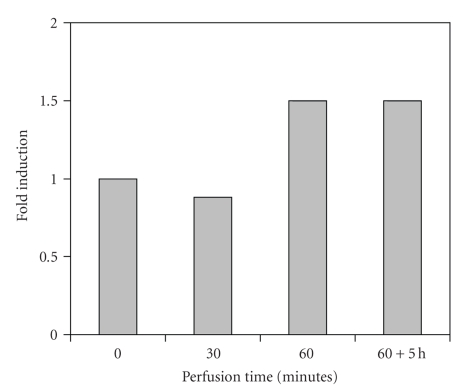
Group III (*n* = 9). HSP72 expression in hyperthermic limb perfusion without cytostatic drug.

**Figure 4:**
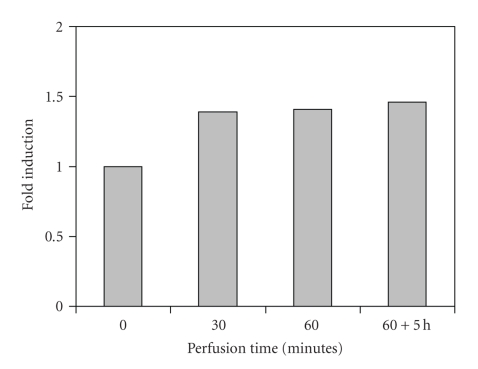
Group IV (*n* = 9). HSP72 expression in hyperthermic limb perfusion with vinblastine.

**Table 1:** Group I (*n* = 12). Gene expression by variation of perfusion time at normothermic conditions without cytostatic drug (gene expression/number of tumor samples).

	Untreated tumor	Perfusion time (minutes)			
		30	60	90	60 + 5 hours
Gene					
*mdr1*	3/3	3/3	3/3	3/3	3/3
mrp1	3/3	3/3	**2/3**	3/3	**2/3**
mrp2	0/3	0/3	0/3	0/3	0/3
lrp	3/3	3/3	3/3	3/3	3/3

**Table 2:** Group II (*n* = 12). Gene expression by variation of perfusion time at normothermic conditions with vinblastine (gene expression/number of tumor samples).

	Untreated tumor	Perfusion time (minutes)			
		30	60	90	60 + 5 hours
Gene					
*mdr1*	3/3	3/3	3/3	3/3	3/3
mrp1	3/3	3/3	3/3	**1/3**	**2/3**
mrp2	0/3	**2/3**	0/3	0/3	0/3
lrp	3/3	3/3	3/3	3/3	3/3

**Table 3:** Group III (*n* = 9). Gene expression by variation of perfusion time at hyperthermic conditions without cytostatic drug (gene expression/number of tumor samples), n.d. = not done (see text).

	Untreated tumor	Perfusion time (minutes)			
		30	60	90	60 + 5 hours
Gene					
*mdr1*	3/3	3/3	3/3	n.d.	3/3
mrp1	3/3	3/3	**1/3**	n.d.	3/3
mrp2	0/3	**2/3**	0/3	n.d.	0/3
lrp	3/3	3/3	3/3	n.d.	3/3

**Table 4:** Group IV (*n* = 9). Gene expression by variation of perfusion time at hyperthermic conditions with vinblastine (gene expression/number of tumor samples), n.d. = not done (see text).

	Untreated tumor	Perfusion time (minutes)			
		30	60	90	60 + 5 hours
Gene					
*mdr1*	3/3	3/3	3/3	n.d.	3/3
mrp1	3/3	3/3	3/3	n.d.	3/3
mrp2	0/3	**1/3**	**1/3**	n.d.	0/3
lrp	3/3	3/3	3/3	n.d.	3/3
